# Role of estuarine habitats for the feeding ecology of the European eel (*Anguilla anguilla* L.)

**DOI:** 10.1371/journal.pone.0270348

**Published:** 2022-07-06

**Authors:** Jérémy Denis, Khalef Rabhi, François Le Loc’h, Frida Ben Rais Lasram, Kévin Boutin, Maria Kazour, Mamadou Diop, Marie-Christine Gruselle, Rachid Amara

**Affiliations:** 1 Université Littoral Côte d’Opale, Université Lille, CNRS, IRD, UMR 8187, LOG, Laboratoire d’Océanologie et de Géosciences, Wimereux, France; 2 IRD, Université Brest, CNRS, Ifremer, LEMAR, Plouzane, France; 3 Parc Naturel Marin des Estuaires Picards et de la Mer d’Opale, OFB, Saint-Étienne-au-Mont, France; National Taiwan University, TAIWAN

## Abstract

This study aims to characterize and compare the feeding ecology of the European eels (*Anguilla anguilla* L.) during the continental phase (i.e. yellow and silver) along a salinity gradient (i.e. lower, middle and upper) in six northern France estuaries (i.e. brackish water). The diet and stable isotopic (i.e. δ^15^N and δ^13^C values) compositions of eels collected with a fyke net in six estuaries (Slack, Wimereux, Liane, Canche, Authie and Somme estuaries) located along the French coast of the eastern English Channel per season over a year were described by combining gut content and stable isotope analyses. Eel guts were dominated by typical BW prey, Malacostraca and Actinopterygii (54% and 40%, respectively), with the gammare *Gammarus zaddachi* and the green crab *Carcinus maenas* (38% and 14%, respectively), and smaller yellow eels of *A*. *anguilla* and juvenile European flounder, *Platichthys flesus* (19% and 14%, respectively) being the most frequently found in their guts. The δ^13^C values of a majority of eels confirmed the sea- and brackish water-specific carbon resources. Dietary and isotopic niche revealed no clear change between total length, silvering stages and seasons, but a significant difference between salinity gradients and estuaries. Eels δ^13^C values showed significant enrichment from upper to lower along the estuaries while the δ^15^N values showed an inverse effect, with the lowest values in the lower part and highest in the upper part. Higher variability in δ^13^C values in larger estuaries suggested that eels feed on a wide range of food sources than in smaller estuaries. While eels in the smaller estuaries fed mainly on Actinopterygii prey, eels in the larger ones had a lower trophic level (i.e. δ^15^N values) and fed mainly on Malacostraca prey. This spatial difference in dietary and isotopic niche is discussed in relation to biological structure of eel and environmental variables.

## Introduction

The European eel (*Anguilla anguilla* L. 1578) is a panmictic [1, 2], a facultative catadromous [3–5] and a long-lived semelparous fish species [3, 5], with a complex life history that occupies a wide variety of habitats between sea-, brackish- and fresh-water (SW, BW and FW, respectively) [6, 7]. The European eels, considered critically endangered due to a drastic decline in their stocks since the 1980s [8], are confronted to numerous anthropogenic pressures (e.g. dams, fishing, pollutions, etc.) encountered in particular during their development phase (i.e. yellow eels) in the SW, BW and/or FW habitats [9]. Yellow eels switch back and forth between SW, BW and FW habitats to feed and grow, or reside in one habitat (i.e. SW, BW or FW) [3]. A significant number of eels may remain in salty water (i.e. SW and BW) all their lives and never live in FW [3, 5, 10]. The behavioural plasticity of yellow eels to use the full range of habitats (i.e. SW, BW and FW) depends on different external factors such as environmental conditions [11, 12], intra- and inter-specific competition [11, 12] and food availability [13]. Nevertheless, SW and BW habitats allow a better growth of yellow eels than FW [14–16], so eels residents in marine and estuarine habitats will be more frequent and beneficial to their development (e.g. [17–19]).

Estuaries are complex and fluctuating environments, known to be important areas for many organisms [20, 21], including diadromous fish. They play an essential role in the life cycle of many fish species as breeding, nursery, feeding and refuge habitats for juveniles and adults [22]. Located at the interface between the marine and continental environment, estuaries are used by diadromous migratory species at various times during their life cycle, as a transit area between sea-, brackish- and fresh-water (SW, BW and FW, respectively), but also as an essential habitat for their development [22, 23]. Currently, there is little information on eels in the BW habitats [24, 25], although they may constitute an important habitat for a significant proportion of the eel population (e.g. [26, 27]). The study of the ecological role of estuarine habitats for resident eels is still extremely limited [24, 25], yet it is necessary to better understand the mechanisms that govern their development and interactions with their environments. This is especially true since the type and quality of habitats influence the development of future breeding adults [4].

The main purpose of the present study is to characterise and compare the feeding ecology of European eels along a salinity gradient in estuarine habitats during their continental development phases. The direct approach of gut content analysis (GCA) [28, 29] and the indirect approach of isotope stable analysis (SIA) [30] are the most common methods in trophic ecology studies. The GCA is an easy way to quickly assess the different prey ingested by a predator. Conversely, the SIA determine the assimilation of prey by the predator, with nitrogen and carbon (i.e. δ^15^N and δ^13^C values) are the most common isotopes measured reflecting respectively the trophic position of the consumers along the food web [31, 32] and the consumption of primary producers [30]. Several studies have investigated the feeding ecology of European eels in estuaries based on GCA [33–35] or SIA [10, 36]. But, few studies combine these two approaches to determine the role of estuaries in the feeding ecology of eels. The combination of GCA and SIA is a robust approach to provide a complete picture of the feeding ecology of a species and improve the interpretation of trophic relationships [37]. The advanced digestion of some ingested prey limits their identification and the short-term (snapshot) view of the diet by GCA requires more detailed examination. SIA of fish muscle validates and complements the trophic relationships elucidated by GCA and provides additional information on sources of primary productivity, habitat use and movement patterns [38]. As sample collection and measurement of stable isotopes of potential prey is often difficult and time consuming, the simultaneous use of GCA and SIA improves the quality of information and makes it faster and easier to elucidate feeding relationships at different spatial and temporal scales.

In this study, taking the northern France estuaries as a case study, the feeding ecology of the eels sampled in estuarine habitats was characterised based on GCA and SIA. The study was carried out using eels collected in 2019 along a salinity gradient (i.e. lower, middle and upper) in six estuaries, located along the French coast in the eastern English Channel. More specifically, this study aimed (i) to assess the diet and stable isotopic (δ^13^C and δ^15^N values) compositions of eels in estuarine habitats, (ii) to compare their dietary and isotopic niche between salinity gradients and estuaries, and (iii) to identify among biological structure of eel (total length and silvering stages) and environmental variables, those that influence their feeding ecology. The biological structure of eel and environmental variables associated with density and diversity of potential eel prey were used as proxies to assess the variability of dietary and isotopic niche. This study contributes significantly to a better understanding of the ecology of European eels, particularly with regard to the use of various aquatic habitats, which has implications for the conservation and management strategies of this critically endangered fish species.

## Materials and methods

### Study area

**Fig 1:**
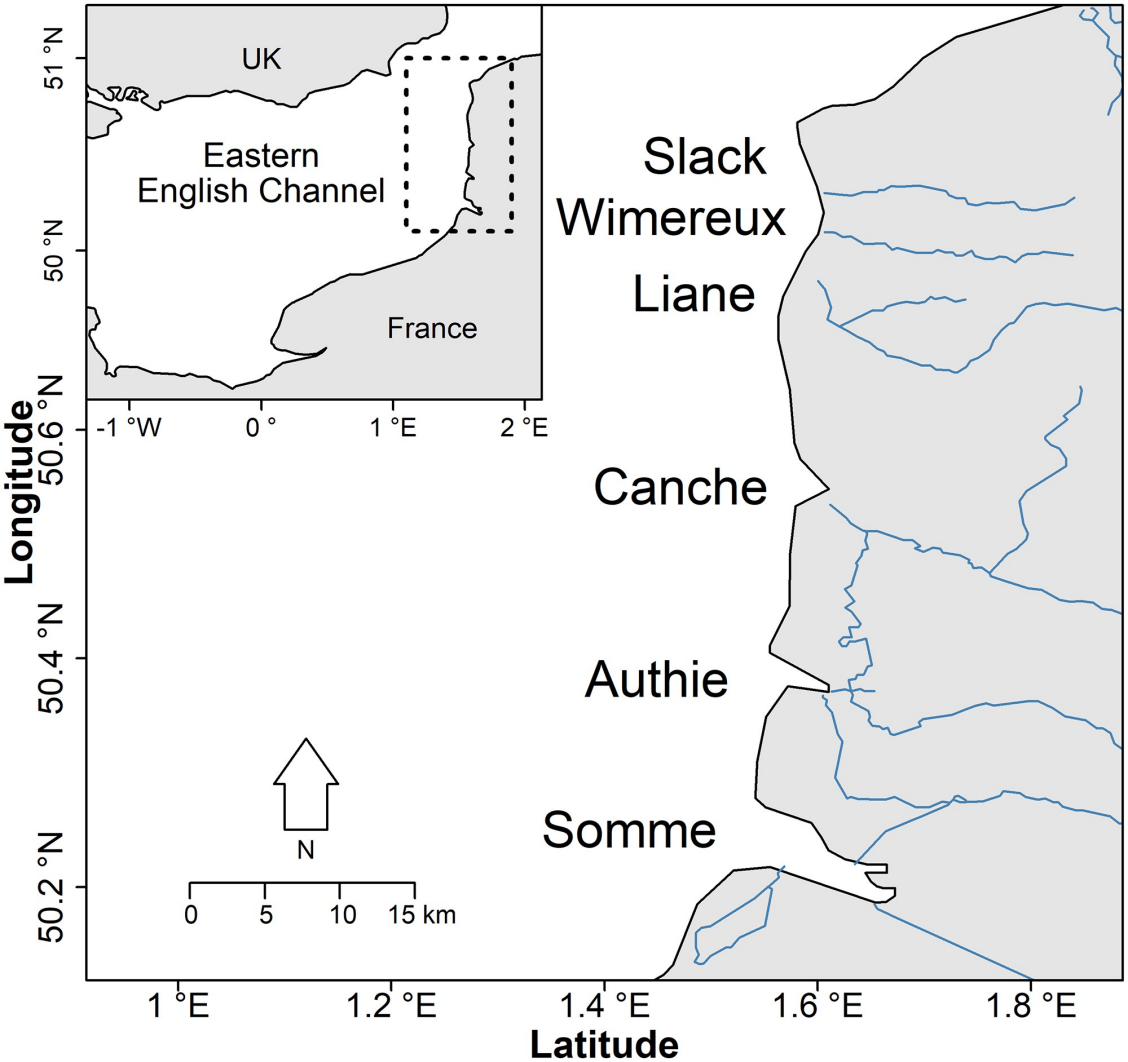
Location of the six estuaries along the French coast in the eastern English Channel.

Sampling was performed in six small and medium estuaries located along the French coast in the eastern English Channel: Slack, Wimereux, Liane, Canche, Authie and Somme estuaries (Fig 1). These estuaries are characterised by a semi-diurnal tide and megatidal regime, with an average tidal range of about 1 m at neap tides and 11 m for the Somme at spring tides [39]. Each of these estuaries has similarities in temperature and salinity ranges but has its own hydro-morpho-sedimentary characteristics in terms of mean flow (water agency hydro.eaufrance.fr; exported in February 2021), surface area (IGN-F maps; exported in February 2021) or entrance width [40]. The Slack and Wimereux, the smallest estuaries, have a mean flow of 0.5 ± 0.1 to 1.2 ± 0.1 m^3^.s^-1^ and a surface area of 13 and 2.2 ha respectively. These two estuaries are much less exposed to tidal actions and sea water entry with an entrance width of 0.02 and 0.04 km, respectively. The Liane, the Canche and the Authie, which are much larger, are characterised by bigger mean flows of 4.1 ± 1.9, 10.7 ± 9.1 and 6.0 ± 5.6 m^3^.s^-1^, and surface area of 222, 340 and 622 ha. They are more exposed to tidal action with entrance widths of 2.7 and 2.9 km for the Canche and Authie, except for the Liane which has infrastructure in the downstream part of the estuary and is more exposed to freshwater inflow. The Somme, the largest of the studied estuaries, with a mean flows of 37.9 ± 33.4 m^3^.s^-1^ and a surface area of 2516 ha, and is the most influenced by sea water with entrance width of 4.6 km. The Slack, Wimereux and Liane are characterised by a bottom type mainly composed of mud, whereas the Canche, Authie and Somme are predominantly composed of sand and muddy sand sediments [41]. The Slack, Liane and Somme are characterised by the presence of dams, delimiting the lower and upper estuaries. The Canche, Authie and Somme estuaries show higher amounts of total nitrogen (8.5 ± 9.7 to 13.7 ± 13.9 mg.L^-1^), are subject to higher human activities (e.g. agriculture, tourism, metal industry, commercial shipping) and can be considered as the most impacted systems of the study area (Naïades database: naiades.eaufrance.fr; exported in February 2021).

### Fish sampling

The permission to collect fish in the estuaries and field site access was issued by the “Préfète de la région Normandie, préfète de la Seine Maritime, Officier de la légion d’honneur, Officier de l’ordre national du mérite, Direction interrégionale de la mer Manche Est-mer du Nord, Service Régulation des Activités et des Emplois Maritimes, Unité Réglementation des Ressources Marines (dram-npe@equipement.gouv.fr): Decision n°196/2019”. In France there is no need for special approval to catch fish by an ethics committee. This study was conducted in accordance with European Commission recommendation 2007/526/EC, on revised guidelines for the accommodation and care of animals used for experimental and other scientific purposes. Eels were anaesthetised with eugenol solution (0.04 ml.L^-1^) before be measured and a total of 121 eels (5 to 6 eels per estuary, station and season) were euthanized with a saturated eugenol solution before being freezed at -80°C for gut content analysis (GCA) and stable isotopes analysis (SIA). The other fish species captured were released alive in the vicinity of the sampling station.

Sampling was performed at each season in March (winter), May (spring), July (summer) and October (autumn) of 2019 at three stations distributed along a salinity gradient (i.e. lower, middle and upper). Eels were collected using fyke nets of 16 m long and a mesh size of 15 mm at the beginning, 10 mm in the middle and 8 mm at the cod end. Two fyke nets were deployed at each station along the shoreline at low tide for a 2 × 24h period.

The total length was measured to the lowest centimetre and their silvering stages were determined by the “silver index” [42] based on the body length (cm), body weight (g), horizontal and vertical eye diameter (mm) and pectoral fin length (mm) measurements. Six silvering stages are be defined with one growth phase (OH), one female growth phase (FII), one female pre-migrant phase (FIII), two female migrating phases (FIV and FV) and one male migrating phase (MII) [43]. In the laboratory, frozen eels were dissected to extract the digestive tract for GCA and a white dorsal muscle was recovered for SIA.

### Gut content analysis

The digestive tract was opened to remove prey from the gut contents and then identified to the lowest possible taxon (using taxonomic keys/references for macrozoobenthos [44, 45], aquatic macroinvertebrate [46] and fish [47, 48] prey) under a binocular microscope and counted. Then, each prey was weighed (in g) using a precision balance (± 0.1 mg). The environmental habitat of each aquatic prey was retrieved from the World Register of Marine Species (WoRMS) and the Catalogue of Life (CoL) databases to assign them as marine (M), marine-brackish (MB), marine-brackish-freshwater (MBF), freshwater (F), in order to determine the habitat origin of the prey according to the water salinity.

The vacuity rate was calculated as the percentage of eels with empty gut and used to estimate the feeding intensity [49]. Diet was described from the relative abundance (*N*), relative weight (*W*) and frequency of occurrence (*FO*) of each prey [29]. The index of relative importance (*IRI*) [50, 51] was calculated to quantify the contribution of each prey taxa to the diet using the following Eq (1):

$$IRI=\left(\%N+\%W\right)\times \%FO$$ (1)

Where %*N* is the percentage of relative abundance, %*W* is the percentage relative weight and %*FO* is the percentage of frequency of occurrence. The *IRI* of each prey taxa was expressed as a percentage (*%IRI*) for summarize the diet composition using the following Eq (2):

$$\%IRI=\;\frac{IRI}{\sum_{i=1}^{n}IRI}\;\times 100$$ (2)

Where *n* is the number of prey taxa *i*.

### Stable isotope analyses

The muscle samples were freeze-dried then ground to a fine powder. As eels are fatty fish, ^13^C-depleted lipids [31] were extracted from the muscle samples using the cyclohexane protocol [52]. Then, the samples were oven-dried at 45°C for 48h and placed in tin cups. The amount of stable isotope carbon (C) and nitrogen (N) was measured using an elemental analyser Flash EA 2000 (Thermo Scientific), connected to an Isotope Ratio Mass Spectrometer (Delta V+) with a ConFlo IV interface (Thermo Scientific) at the Pôle Spectrométrie Océan in Plouzané, France. Results are expressed as δ (delta) notation relative in parts per mile (‰) using the following Eq (3):

$$\delta x=\left[\left(\frac{R_{sample}}{R_{standard}}\right)-1\right]\;\times 1000$$ (3)

Where δx is δ^13^C or δ^15^N values (‰), R_sample_ is the ratio ^13^C/^12^C or ^15^N/^14^N and R_standard_ is value based on the Pee Dee Belemnite for carbon or atmospheric Nitrogen for nitrogen. The calculated uncertainties on the repeated measurement of the acetanilide internal standards were of experimental precision <0.3‰ for δ^13^C and δ^15^N values. The C/N ratios above 4 indicating that the tissues have high levels of lipid to bias the δ^13^C values were excluded from the analyses.

The trophic position (TP; Cabana and Rasmussen, 1996) [53] of eels was calculated following Eq (4):

$$TP=TP_{Base}+\frac{\delta^{15}N\;-\;\delta^{15}N_{Base}}{TDF}$$ (4)

Where TP_Base_ is the trophic position of isotopic baseline, δ^15^N is the nitrogen isotopic of eels, δ^15^N_Base_ is the isotopic nitrogen of the baseline, and TDF is the trophic discrimination factor. Mean δ^15^N values of sediment organic matter (SOM) inside the Canche (between 4.67 to 7.19‰) [54] and the Somme (between 6.93 to 8.98‰) [55] estuaries at different salinity and seasons (S1 Table) were used as baseline resource to calculate the δ^15^N_Base_. The baseline resource used was considered as a TP set at 1 and the TDF has been set to 3.4‰ [56].

Layman metrics [57] were calculated to estimate eels isotopic niche widths using their δ^13^C and δ^15^N values. Isotopic niche widths were estimated using a Bayesian approach based on multivariate ellipse-based metrics [58]. The convex hull area (TA) represents the width of the total isotopic niche of a population [57], and the standard ellipse area (SEA) is a bivariate measure of mean isotopic niche for all individuals [58]. The SEA can be underestimated when samples are small and should therefore be corrected using the SEAc metric [58]. The analyses were performed using the *SIBER* [58] package in R.

The trophic positions and δ^13^C values of eels will be combined and compared with the composition of gut contents and the origin of ingested prey to explore variations in feeding habitats along a salinity gradient (δ^13^C values differ along salinity gradients [38]) and the dependence of eels on certain categories of prey (e.g. benthic macroinvertebrates rather than fish [10]).

### Statistical analyses

As the data did not comply with the parametric assumption of normality (Shapiro-Wilk test) and homoscedasticity of variance (Levene’s *F* test), total length of eels was compared with nonparametric Kruskall-Wallis test. Dunn test was used for post hoc comparisons. The percentage of silvering stages between six estuaries were compared with Chi-square test. The Shapiro–Wilk test, Levene’s *F* test, Kruskall-Wallis test, Dunn test and Chi-square test were performed using the *Stats* package in R.

The effects of total length, silvering stages, salinity gradients, estuaries and seasons on the diet and stable isotope (δ^13^C and δ^15^N values) compositions of eels were tested by a permutational multivariate analysis of variance (PERMANOVA) based on the Bray-Curtis or Euclidean distance [59]. Pairwise tests were used to examine differences between factors when the PERMANOVA indicated significant main effects, and p-values were adjusted using False Discovery Rate [60]. The PERMANOVA and pairwise tests were performed using the *vegan* [61] and the *RVAideMemoire* [62] packages in R.

The spatial dietary similarities were explored using non-metric multidimensional scaling (nMDS). A hierarchical classification analysis (HCA) based on Bray–Curtis similarity matrix from %IRI of prey categories found in the eel gut contents was carried out in order to group the salinity gradients (i.e. lower, middle and upper) and estuaries with similar eel diet. The number of groups was selected from the group-average sorting and comparison made by similarity profile test (SIMPROF). These groups were represented by an ordination plot using nMDS with Bray–Curtis distance calculated by groups. One-way analysis of similarity (ANOSIM) based Bray–Curtis dissimilarity matrix was then performed to compare their groups with similar diets. Similarity percentages (SIMPER) were used to determine which prey taxa (i.e. accounted for 80% of the similarity) contributed to average similarity within a group and to provide measures of the relative dissimilarity among groups [63]. The HCA, SIMPROF, nMDS, ANOSIM and SIMPER were performed using the *vegan* [61], *clustsig* [64] and *Stats* packages in R. Schoener diet overlap index (SDOI) [65] based on the %IRI of prey categories was calculated to estimate the percentage similarity of diets between salinity gradients and estuaries according to the following Eq (5):

$$\propto \;=1-0.5\;\left(\;\sum_{i=1}^{n}\left|P_{xi}-\;P_{yi}\right|\right)$$ (5)

Where *α* is the dietary overlap, *P*_*xi*_ and *P*_*yi*_ are proportions of prey taxa *i* (*%IRI*) between groups *x* and *y* (i.e. between salinity gradients and estuaries) and *n* is the total number of prey taxa. Dietary overlap index is considered significant for values exceeding 0.6 (≥60%) [66, 67].

The spatial overlaps of isotopic niche region were estimated by a probabilistic method that uses stable isotopes values [68]. This probabilistic method calculates niche regions and pairwise niche overlap without considering sample size. The δ^13^C and δ^15^N values for each salinity gradient (i.e. lower, middle and upper) and estuary were used to calculate the average overlap between niche regions with 95% Bayesian intervals based on 10,000 iterations in bivariate dimension. Uncertainty was estimated using a Bayesian framework considering the sample size [68]. The isotopic niche region overlaps were performed using the R package *nicheROVER* [69].

The influence of biological structure of eel and environmental variables on the dietary and isotopic niche was explained using redundancy analysis (RDA). The RDA was performed as a constrained ordination technique to determine how the spatial difference of dietary (%IRI of prey categories, and percentage of marine-brackish and freshwater prey) and isotopic niche (δ^13^C values, TA, SEAc, and TP) of 121 eels analysed could be explained by biological structures of eel and environmental variables. The two biological structures of eel used were the total length and silvering stages which influence the range of prey size and ontogenetic change in the diet with increasing body size (e.g. [36, 70]). The four environmental variables selected are the surface area of the estuary to determines the diversity of habitats present (e.g. [71, 72]), the entrance width which reflects the connectivity of the system with the marine environment and access to the estuary for marine and diadromous species (e.g. [40]), and the sediment types which are known to affect the distribution of estuarine macrobenthos (e.g. [41, 73, 74]). The nitrogen concentration is used as indicators of anthropogenic pollutions that can influence the δ^15^N values of eels [75]. The data were normalized (log-transformed), then centred and reduced before analyses. Biological structures of eel and environmental variables were significantly selected using a Monte Carlo permutations test (*n* = 999) [76]. Contribution of each selected co-variable to diet and isotopic niche variation was finally assessed using a variance partitioning analysis and a permutation test [76]. The RDA and variation partitioning were performed using the *vegan* [61] packages in R.

## Results

### Eels samples for gut contents and stable isotopes analyses

The total length of the 121 analysed eels ranged between 260 and 924 mm. The mean size of individuals showed no significant differences between salinity gradients of six estuaries (Kruskall-Wallis test, p = 0.39) (Table 1). The percentage of eels per silvering stage did not vary significantly between the six estuaries (Chi-square test, p = 0.31). Regardless of the estuary, most of the eels were in the yellow stage (75–95%) and only 5–25% in the silver stage. Half of the individuals were of undetermined (OH) while the other half (40%) had a sex ratio dominated by females. Males were slightly more abundant only in the Slack, Wimereux and the Liane estuaries. The Wimereux and the Liane estuaries had 25% of silver eels (i.e. FIV, FV and MII), which is higher than in the other estuaries (between 4.8 and 15%) (Table 1).

**Table 1:** Number of individuals analysed for gut content and stable isotope analysis along salinity gradient (i.e. lower, middle and upper) in the six estuaries, and their mean total length (mm) ± standard deviation, and percentage of individuals by silvering stages (OH growth phase, FII female growth phase, FIII female pre-migrant phase, FIV and FV female migrating phases and MII male migrating phase).

Estuary	Number of individuals			Total length (mm)			Silvering stage (%)					
	Lower	Middle	Upper	Lower	Middle	Upper	OH	FII	FIII	FIV	FV	MII
**Slack**	6	3	11	419.3 ± 138.5	473.7 ± 107.6	395.2 ± 85.7	60.0	10.0	15.0	-	-	15.0
**Wimereux**	6	6	8	457.5 ± 167.5	424.7 ± 102.1	454.1 ± 107.8	35.0	5.0	35.0	-	-	25.0
**Liane**	2	14	4	509.5 ± 94.0	505.0 ± 148.1	560.5 ± 167.9	30.0	30.0	15.0	10.0	5.0	10.0
**Canche**	4	7	7	462.3 ± 178.7	377.7 ± 84.5	387.3 ± 84.2	66.7	11.1	11.1	-	5.6	5.6
**Authie**	5	12	4	510.6 ± 126.9	434.8 ± 61.3	364.0 ± 89.1	57.1	28.6	9.5	-	4.8	-
**Somme**	5	4	11	472.8 ± 89.6	499.3 ± 262.8	502.5 ± 183.1	50.0	20.0	15.0	10.0	-	5.0

### Diet composition and stable isotopic values of eels

Of the 121 eels, 22% had an empty gut (Table 2). The vacuity rates varied between the salinity gradients, with the highest rates recorded in the upper part, mainly in the Slack and Somme estuaries (13%), and in the middle part, mainly in the Liane and the Authie estuaries (9%), while in the lower part were below 3%. The vacuity rates were higher in the Slack (30%), the Liane (25%) and the Somme (24%) estuaries, while in the other estuaries the vacuity rates did not exceed 20%. In total, 32 prey taxa were identified in the eel gut contents (Table 2). The majority of the prey was typical of brackish water (BW) habitats, with 42% from marine-brackish-freshwater habitat (MBF), 38% from marine-brackish habitat (MB) and 16% from marine habitat (M) (Table 2). Freshwater prey (F) represented barely 4% of the diet. The diet showed an important diversity of prey consumed by the eels, mainly dominated by Malacostraca and Actinopterygii which were respectively the most important prey categories in terms of percentage index of relative importance (%IRI) with 54% and 40% of the diet. The diet composition of the eel was not significantly different for total length (PERMANOVA, Pseudo-F_5, 93_ = 0.7414, p = 0.658), silvering stages (PERMANOVA, Pseudo-F_5, 93_ = 1.3615, p = 0.182), salinity gradients (PERMANOVA, Pseudo-F_2, 93_ = 0.5418, p = 0.709) and seasons (PERMANOVA, Pseudo-F_3, 93_ = 1.2883, p = 0.265). However, their diet varied significantly between estuaries (PERMANOVA, Pseudo-F_5, 93_ = 6.5205, p < 0.001). Among the Malacostraca prey, the gammare *Gammarus zaddachi* and the green crab *Carcinus maenas* had the highest %IRI in all the collected samples representing respectively 38% and 14% of the total identified prey. These prey were the most abundant in the diet of eels collected from the Canche (60 and 10%, respectively), the Authie (80 and 16%, respectively) and the Somme (5 and 35%, respectively) estuaries (Table 2). For Actinopterygii prey, smaller yellow eels of *A*. *anguilla* and juvenile young-of-the-year European flounder, *Platichthys flesus* were the most important prey representing respectively 19% and 14% of the total gut contents. Smaller yellow eels were found in the gut contents mainly in the Liane (67%) and the Wimereux (30%) estuaries, while *P*. *flesus* was found mainly in the Wimereux (50%) and the Slack (42%) estuaries. A diversity of insects was found in the gut contents of eels, particularly in the Slack (20%) and the Wimereux (16%) estuaries, with mainly *Corixa* sp. and Calliphoridae and Chironomidae larvae. Regarding other prey categories, the Polychaeta *Hediste diversicolor* represented up to 29% of the gut contents in the Somme estuary and the Gastropod *Stephonysa marmorata* represented up to 26% in the Liane estuary. Other prey had a low %IRI of less than 3% of total gut contents (Table 2).

**Table 2:** Prey composition observed in the gut contents of European eels collected in the six estuaries. Percentage values of prey occurrence (%F), abundance (%N), weight (%W), index of relative importance (%IRI) and empty guts (%) are indicated.

Prey taxa	Env	%F	%N	%W	%IRI						
					Slack	Wimereux	Liane	Canche	Authie	Somme	Total
Polychaeta											2.44
*Arenicola marina*	M	1.06	0.06	0.13				0.10			0.01
*Hediste diversicolor*	MBF	6.38	4.05	4.72	0.68	0.67			0.26	29.62	2.43
Arachnida											0.04
*Argyroneta aquatica*	F	2.13	0.17	0.03		1.31					0.02
*Dolomedes* sp.	F	1.06	0.28	0.05	1.36						0.02
Insecta											1.62
Haliplidea larvae	F	3.19	0.50	0.01	0.25		0.41				0.07
Calliphoridae larvae	F	1.06	1.61	0.09		5.98					0.08
Chironomidea larvae	F	8.51	2.16	0.32		7.89	1.52	0.03		0.42	0.92
Chironomidea pupae	F	4.26	0.44	0.06	1.02		0.36				0.09
Unid. Tipulidea	F	1.06	0.06	<0.01					0.03		<0.01
*Corixa* sp.	F	3.19	2.05	0.14	18.58		0.03				0.30
Lepidoptera larvae	F	1.06	0.17	0.09		0.76					0.01
Crambidea larvae	F	1.06	0.06	0.01		0.22					<0.01
*Tettigonia* sp.	T	1.06	0.06	0.05		0.29					0.01
Unid. Taeniopterygidea	F	1.06	0.06	<0.01				0.03			<0.01
Trichoptera larvae	F	1.06	0.06	0.01			0.03				<0.01
Limnephilidea larvae	F	1.06	0.22	0.05		0.90					0.01
Insecta eggs	F	1.06	2.77	0.01						6.59	0.13
Malacostraca											53.75
*Corophium volutator*	M	1.06	0.50	0.04						1.24	0.02
*Gammarus zaddachi*	MB	26.6	23.95	8.85	2.92	0.93		60.34	80.09	5.30	37.84
*Carcinus maenas*	M	26.6	2.94	8.96	9.79		0.56	9.66	15.65	34.96	13.73
*Crangon crangon*	M	7.45	1.05	3.71	1.07			6.37	0.97		1.54
*Palaemon elegans*	M	3.19	0.44	3.98					0.14	12.19	0.61
*Gnathia* sp.	MB	2.13	0.11	<0.01						0.53	0.01
Actinopterygii											39.63
Unid. Actinopterygians		3.19	0.17	0.37					0.38	0.30	0.07
*Anguilla anguilla*	MBF	10.64	2.99	37.72		30.19	66.88	1.37		6.86	18.79
*Sprattus sprattus*	MB	1.06	0.39	0.80				0.65			0.05
*Gasterosteus aculeatus*	MBF	1.06	0.06	0.41			0.26				0.02
*Pomatoschistus microps*	MBF	12.77	2.27	9.50	22.40	0.95	1.19	4.81	2.47	0.71	6.52
*Platichthys flesus*	MBF	22.34	4.77	9.63	41.92	49.89	1.50	16.64		1.13	13.95
Eggs		3.19	1.66	0.03			1.48			0.13	0.23
Bivalvia											0.02
*Limecola balthica*	M	3.19	0.17	0.01			0.25				0.02
Gastropoda											2.48
*Stenophysa marmorata*	F	1.06	43.79	10.23			25.54				2.48
**Vacuity rate (%)**					30.0	20.0	25.0	15.8	19.1	23.8	22.3

The environmental habitats (Env.) of each prey with marine (M), marine-brackish (MB), marine-brackish-freshwater (MBF), freshwater (F) and terrestrial (T) is also indicated.

Stable isotopes of eels ranged between -32.6 to -15.1‰ for δ^13^C values; whereas for the δ^15^N values ranged between 11.7 to 19.0‰. The majority of eels analysed showed δ^13^C values reflecting SW and BW-specific carbon resources. The diet does not differ significantly between total length (PERMANOVA, Pseudo-F_4, 118_ = 1.2638, p = 0.258) and seasons (PERMANOVA, Pseudo-F_3, 118_ = 1.7676, p = 0.139). The PERMANOVA indicated a low significant effect of silvering stages on δ^13^C and δ^15^N values (PERMANOVA, Pseudo-F_5, 118_ = 2.7383, p = 0.017). The δ^13^C and δ^15^N values of eels were similar between silvering stages, except for females in the FIV stage (Pairwise PERMANOVA, p < 0.05), but this may be related to the small number of individuals analysed (Table 1). However, there was a high significant effect between salinity gradients (PERMANOVA, Pseudo-F_2, 118_ = 18.4892, p < 0.001) and estuaries (PERMANOVA, Pseudo-F_5, 118_ = 6.1809, p < 0.001) reflecting a spatial difference in isotopic niche. Eels δ^13^C values showed significant enrichment along the estuaries from upper (-28.2 ± 1.0 to -24.7 ± 2.5‰) to lower (-26.0 ± 0.1 to -17.3 ± 0.8‰) part. The PERMANOVA revealed a salinity gradient effect on eels δ^15^N values, with the highest mean values in the lower part (14.3 ± 0.8 to 18.5 ± 0.5‰) and lowest in the upper part (12.8 ± 0.5 to 16 ± 1.6‰). The eels collected in the Slack, the Wimereux and the Liane estuaries showed higher mean δ^15^N values (15.5 ± 1.7 to 18.5 ± 0.5‰) than that caught in the other estuaries (12.8 ± 0.5 to 15.5 ± 0.4‰) (Table 3). This may reflect a difference in diet in favour of more nitrogen enriched prey in eels in smaller estuaries and the consumption of more nitrogen depleted prey in larger estuaries, as shown by their trophic positions (TP) (Table 3). Mean eels δ^13^C values were higher for the Canche, Authie and Somme estuaries (-23.8 ± 2.5 to -17.3 ± 0.8‰), indicating a more marine carbon source in the larger estuaries compared to the smaller estuaries (-26.8 ± 0.7 to -22.3 ± 1.3‰). The results of total and standard ellipse areas (TA and SEAc) reveal a larger isotopic niche in the Canche (10.4% and 19.4%, respectively) and the Somme (10.8 to 37.3% and 20.4 to 20.5%, respectively) compared to the Slack, Wimereux, Liane and Authie estuaries (between 0.1 to 8.7% of TA and 0.5 to 5.2% of SEAc) (Table 3).

**Table 3:** Isotopic metrics with mean ± standard deviation of δ^13^C and δ^15^N (‰) of European eels along salinity gradient (i.e. lower, middle and upper) in the six estuaries and total convex hull area (TA; %), corrected standard ellipse areas (SEAc, %), and trophic position (TP).

Estuary	δ^13^C (‰)			δ^15^N (‰)			TA (%)			SEAc (%)			TP		
	Lower	Middle	Upper	Lower	Middle	Upper	Lower	Middle	Upper	Lower	Middle	Upper	Lower	Middle	Upper
**Slack**	-22.3 ± 1.3	-23.6 ± 1.7	-24.7 ± 2.5	16.3 ± 0.5	16.4 ± 1.2	15.6 ± 0.7	2.8	0.1	8.7	2.5	0.5	5.2	4.0	3.7	4.0
**Wimereux**	-24.0 ± 1.3	-25.0 ± 1.2	-25.3 ± 1.2	16.4 ± 0.7	15.5 ± 1.7	16.0 ± 1.6	2.9	4.1	5.8	2.9	4.4	5.2	4.3	3.6	4.0
**Liane**	-26.0 ± 0.1	-26.8 ± 0.7	-26.4 ± 0.6	18.5 ± 0.5	16.9 ± 1.6	15.7 ± 1.9	-	8.1	0.9	-	4.0	1.6	5.1	4.0	4.0
**Canche**	-23.1 ± 5.5	-22.5 ± 1.8	-23.8 ± 2.5	14.3 ± 0.8	14.5 ± 0.9	13.4 ± 0.6	10.4	4.7	5.0	19.4	3.8	4.0	3.7	3.3	3.3
**Authie**	-18.5 ± 2.2	-22.8 ± 2.6	-28.2 ± 1.0	15.1 ± 0.3	14.6 ± 0.6	12.8 ± 0.5	1.7	4.1	0.7	2.1	2.5	1.5	3.7	3.5	3.2
**Somme**	-17.3 ± 0.8	-21.4 ± 6.5	-28.0 ± 5.0	15.5 ± 0.4	14.8 ± 0.7	14.3 ± 1.2	0.8	10.8	37.3	1.0	20.4	20.5	3.2	3.3	2.8

### Spatial similarities of dietary and isotopic niche

**Fig 2:**
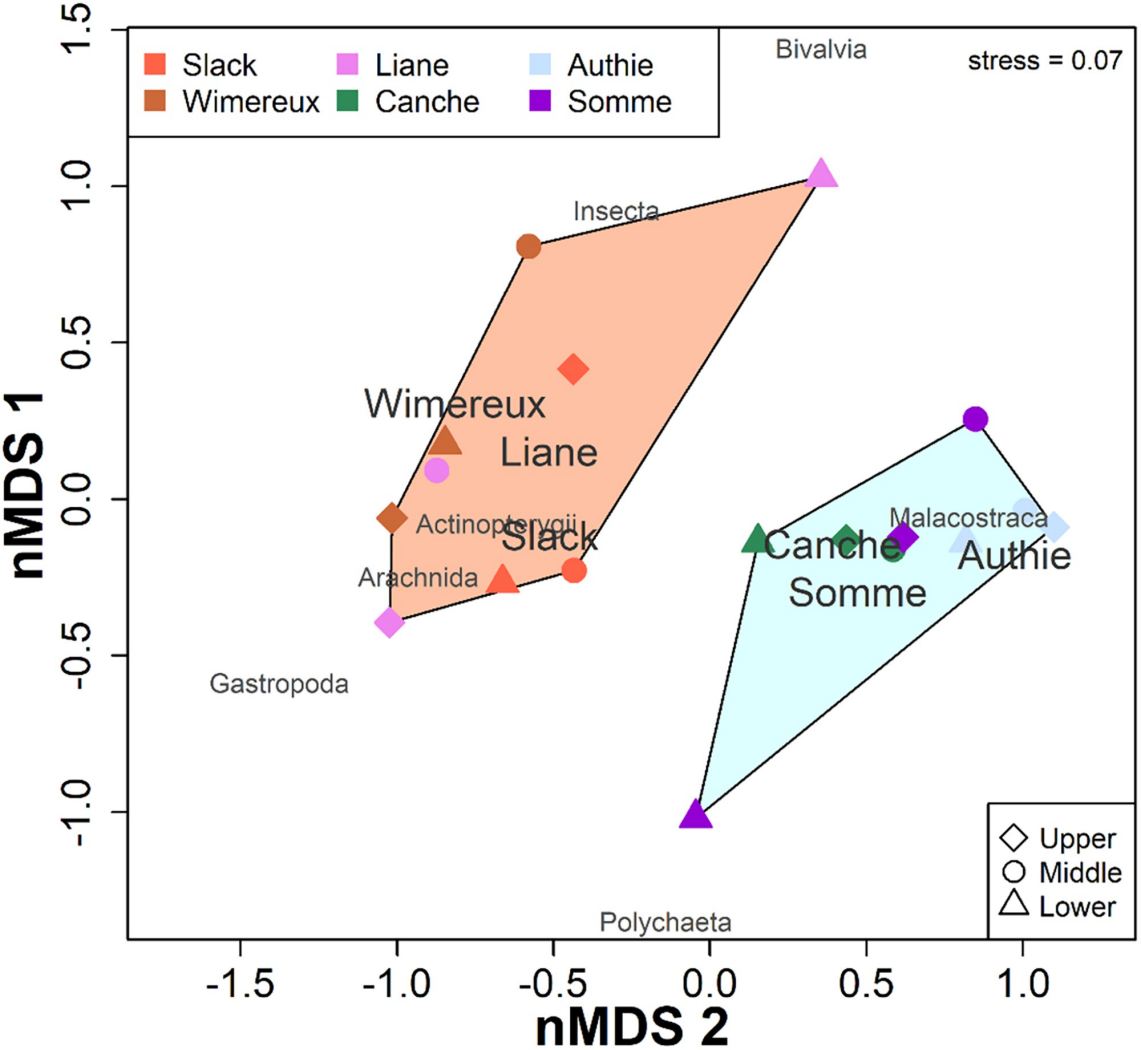
Two-dimensional nMDS ordination performed on the index of relative importance (%IRI) of prey categories in the gut contents of European eels collected along salinity gradient (i.e. lower, middle and upper) in the six estuaries. The ellipses represent the two groups of estuaries identified in the HCA. Stress value is indicated in the top right.

According to the hierarchical classification analysis (HCA) and the similarity profile test (SIMPROF) based on %IRI of prey categories between salinity gradients and estuaries, two distinct groups of estuaries were identified and distributed along the two ordinations of the non-metric multidimensional scaling (nMDS) (Fig 2, stress value = 0.07). These groups were significantly different with average dissimilarity of 75% (ANOSIM, R = 0.75, p<0.001) and indicates clearly that the whole diet differs between estuary groups but not between salinity gradients. The prey categories that determine the dissimilarity between groups are the Malacostraca, Actinopterygii and Insecta accounting for over 91% of the dissimilarity (SIMPER, p<0.01). The first group including the Slack, the Wimereux and the Liane estuaries, was discriminated by a similar diet composed mainly of Actinopterygii (dissimilarity of 32%) and Insecta (dissimilarity of 15%), while the diet composition for the second group associating the Canche, the Authie and the Somme estuaries, was mainly composed of Malacostraca (dissimilarity of 44%). The Schoener diet overlap index (SDOI) indicated a diet overlap of 67 to 85% between the Slack, the Wimereux and the Liane estuaries (i.e. the first group) and of 73 to 83% for the Canche, the Authie and the Somme estuaries (i.e. the second group) (Table 4). The variations in the diet composition of eels between salinity gradients were low (with an overlap of 61 to 95%; Table 4), except for the Liane and the Somme estuaries which show a different diet in the upper part (with an overlap of 0 to 38%; Table 4), due to a dominance in Bivalvia in the Liane estuary and Polychaeta prey in the Somme estuary (Fig 2).

**Table 4:** Fish dietary overlap calculated with the Schoener diet overlap index (SDOI; %) and isotopic niche region overlap (%) estimates with 95% Bayesian credible intervals of European eels along salinity gradient (i.e. lower, middle and upper) in the six estuaries.

	Estuary	Station			Estuary					
		Lower-Middle	Lower-Upper	Middle-Upper	Slack	Wimereux	Liane	Canche	Authie	Somme
**SDOI**	**Slack**	**84**	**62**	**62**	100	**85**	**67**	46	19	35
	**Wimereux**	**61**	**79**	45		100	**72**	31	4	20
	**Liane**	18	0	**66**			100	30	4	18
	**Canche**	**80**	**88**	**92**				100	**73**	**83**
	**Authie**	**91**	**86**	**95**					100	**73**
	**Somme**	17	38	**79**						100
**Isotopic niche region overlap**	**Slack**	18	50	27	100	**66**	28	38	20	54
	**Wimereux**	54	56	**71**		100	39	33	18	41
	**Liane**	0	0	43			100	15	8	25
	**Canche**	44	43	48				100	**72**	**72**
	**Authie**	36	0	14					100	**62**
	**Somme**	36	0	14						100

Bold characters indicate significantly high values (>60%).

**Fig 3:**
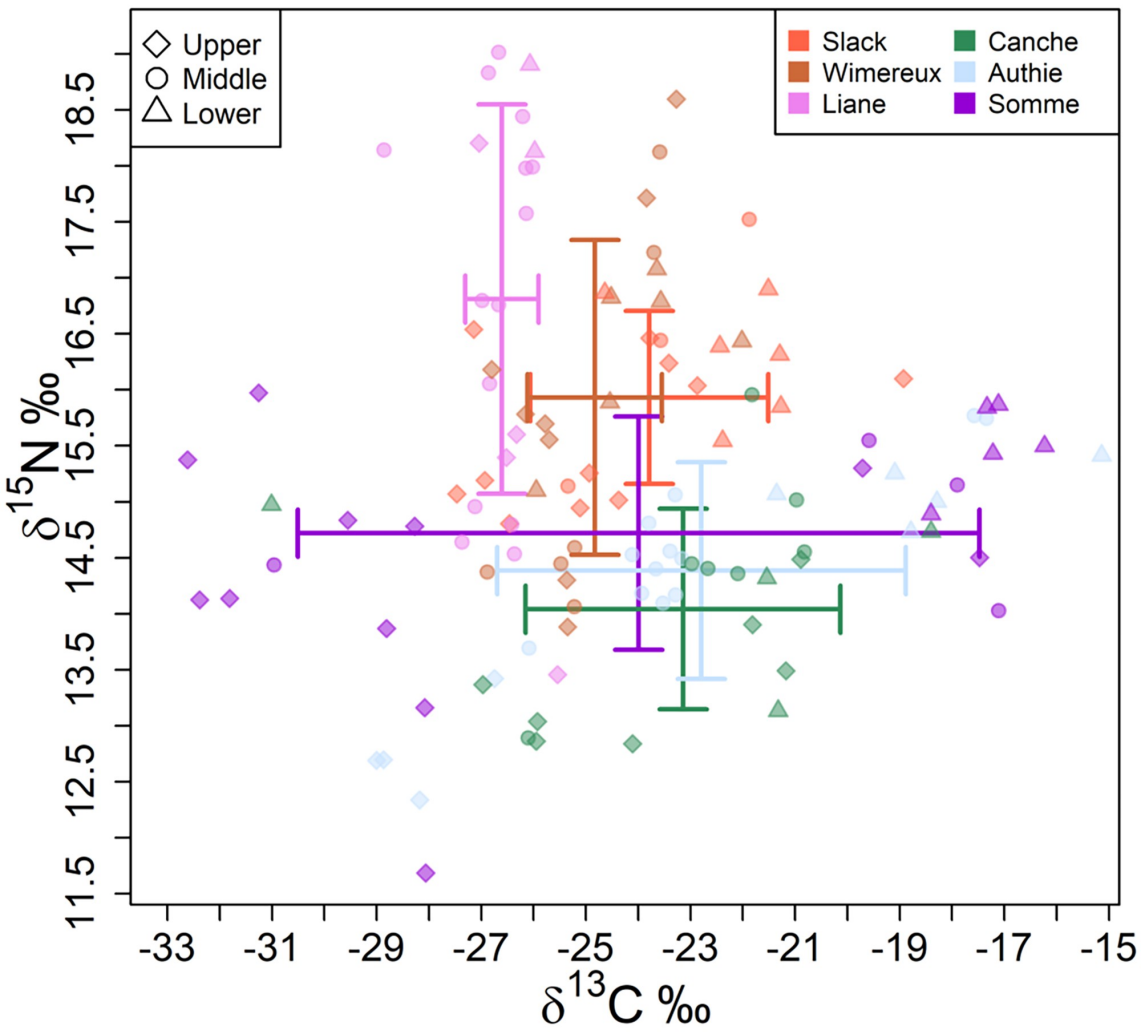
δ^13^C and δ^15^N values (‰) of European eels collected along salinity gradient (i.e. lower, middle and upper) in the six estuaries. The mean ± standard deviation of the δ^13^C and δ^15^N values for the six estuaries is also represented.

The same results were obtained for the SIA. The probabilistic niche region indicated that the isotopic overlap between eels in the Slack and Wimereux estuary is 66%, and from 62 to 72% between the Canche, Authie and Somme estuary (Table 4). In contrast to the GCA, no isotopic niche overlap was found between salinity gradients (Table 4), suggesting a variation in baseline carbon resources according to position in the estuary (i.e. lower, middle and upper) rather than a difference in feeding (Fig 3).

### Environmental and biological influence on dietary and isotopic niche

**Fig 4:**
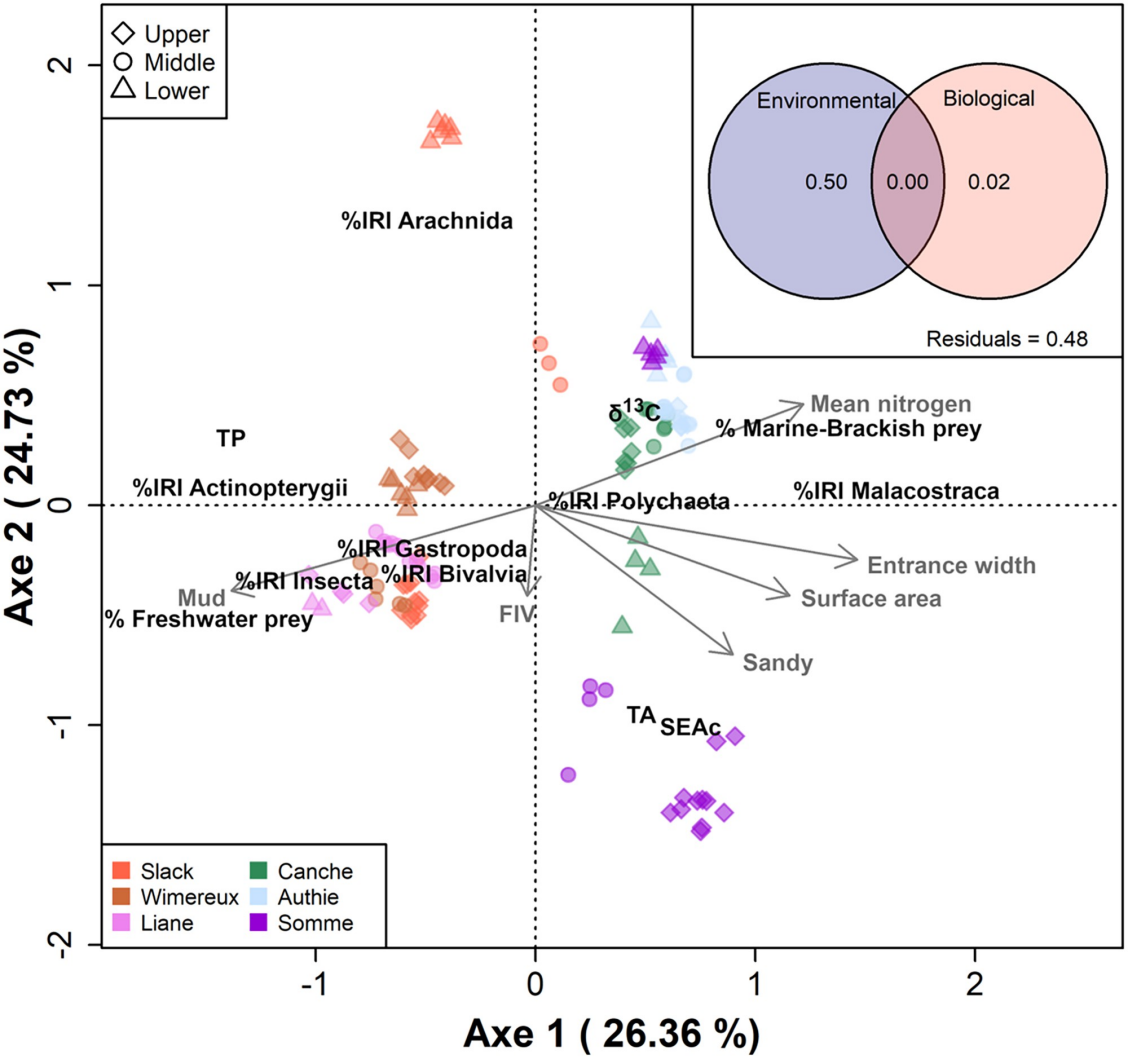
Redundancy and variance partitioning (top-left) analyses on the dietary (index of relative importance (%IRI) of prey categories (see Table 2), and percentage of marine-brackish and freshwater prey) and isotopic niche (δ^13^C, total convex hull area (TA), corrected standard ellipse areas (SEAc), and trophic position (TP)) of European eels along salinity gradient (i.e. lower, middle and upper) in the six estuaries constrained by selected biological structures of eel (silvering stages: FIV female migrating phases) and environmental variables (surface area, sediment types, entrance width and mean total nitrogen). Numbers in the circles (top-left) represent the proportion of variance explained by each co-variable.

RDA was applied to analyse the eel spatial dietary and isotopic niche variation in response to biological structures of eel and environmental variables (Fig 4). The selected co-variables explained 52% (adjusted *r*^*2*^) of the total variance. Two first axes of the RDA explained 51.1% of the total variance in the dietary and isotopic niche was significantly different according to the surface area, entrance width, sediment types and mean total nitrogen. The diet and isotopic niche of eels from the Canche, the Authie and the Somme estuaries characterized mainly of SW and BW prey (Malacostraca and Polycheata) and higher δ^13^C values, TA and SEAc were associated with higher surface area, entrance width and mean total nitrogen and sandy sediment. The second group of eels included individuals belonging to the Slack, the Wimereux and the Liane estuaries characterised by a diet mainly composed of freshwater prey (Actinopterygii, Arachnida, Gastropoda, Bivalvia and Insecta), and higher TP were associated with a muddy sediment less influenced by the sea. The variance partitioning analysis showed the main contribution of environmental variables (50%) to the explained variation of dietary and isotopic niche (51%), followed by biological variables (2%) (Fig 4).

## Discussion

### Estuarine eels feeding ecology

In previous studies, the gut content analysis (GCA) of yellow and silver European eels confirmed that they mainly feed on Malacostraca and Actinopterygii prey [10, 33–35, 77] which is in accordance with the eels analysed in the present study (Table 3). We found a lower vacuity rate (< 30%) compared to other studies like in the Tagus estuary (32–41%) [33] and in Lough Corrib (43–61%) [35]. This vacuity rate is probably overestimated since the use of fyke nets could lead to an overestimation of the eel diet due to the long time (i.e. 24h) between the laying and the hauling of the nets. However, the low percentage of empty gut in the present study could be explained by the passive capture of eels by fyke nets, which reduces the stress and the possibility of regurgitating gut contents [78]. As in previous studies [33, 77], Malacostraca was the dominant prey taxa (54% of IRI) in the diet of European eels caught in the studied estuaries. The Malacostraca prey included the crab *C*. *maenas* and the gammare *G*. *zaddachi*. Similarly, in the Tagus estuary in Portugal [33] and the Gironde estuary in France [77], the eels fed on the crustaceans, specifically amphipods, crabs and shrimps, to be dominant prey for European eels captured in the upper part in the Severn estuary in UK the eels fed on other Malacostraca prey, with the shrimp *Crangon crangon* and Mysidacea *Neomysis integer* being the most important prey species in terms of biomass [79]. Actinopterygii was the second dominant prey taxa in %IRI (40%), consisting mainly of two fish species that frequent BW habitats such as estuaries with a resident juvenile European flounder *P*. *flesus*. A significant amount of smaller eels *A*. *anguilla* (19%) were found in the gut contents. Both field and experimental studies have reported cases of cannibalism in several eel species [80–82]. Insecta, Gasteropoda and Polychaeta prey were the most widespread other prey taxa, with low %IRI values below 3%. The high diversity of prey recorded in the gut contents confirms the opportunistic character of European eels, particularly in feeding on benthic prey [83–85].

Stable isotope analyses (SIA), based on both stable carbon and nitrogen isotopes (δ^13^C and δ^15^N values), indicated that European eels fed across several basal food sources and trophic positions (TP). SIA is an approach to assess the origin of food sources, the TP of species and trophic interactions between species [86], and also to determine the movements between feeding habitats along contrasting salinity gradient in diadromous fishes over a short-term of several weeks or months [10, 36]. Yellow and silver eels had broad δ^13^C values (-32.6 to -15.1‰) suggesting differences in baseline carbon resources. However, the majority of eels showed low variations in δ^13^C values (Table 3 and Fig 3) between individuals within the same salinity zones suggesting minimal movement between the salinity gradients (i.e. lower, middle and upper). The European eels’ δ^15^N values (11.7 to 19.0‰) indicated a variation of TP approximately 3.4 to 4.2, based on the trophic discrimination factor of 3.4‰ [56]. Our results support the hypothesis that eels are able to feed at different TP in BW habitats such as estuaries [36], and thus benefit from a wider range of potential food sources offered by BW habitats. Indeed, BW habitats support a high trophic diversity of macrozoobenthos and fish, including SW and FW species that use the estuaries as a breeding or a nursery area during their life cycle [39, 87].

The δ^15^N values can be used as a tracer of total nitrogen inputs from untreated domestic, industrial and/or agricultural activities assimilation into the food web by primary producers [38, 75]. In the Canche and the Somme estuary, SOM δ^15^N values (S1 Table) were rather similar along the salinity gradient during the same season, indicating a relatively low input of total nitrogen from human catchment activities. Our results also show that despite high total nitrogen values in the larger estuaries, eels had the lowest δ^15^N values compared to eels in the smaller estuaries, which confirms a weak influence of anthropogenic pollution.

The European eels, considered as an opportunistic feeder [83, 85, 88], can change its diet depending on various factors, including the silvering stages, total length [89–91], weight [10] or head morphology [70, 92, 93]. During ontogeny, the young eels feed on invertebrates, then expand their range of prey size with increasing body size for feeding almost exclusively on fish, and thus optimise energy intake by consuming prey at higher trophic positions [70, 94, 95]. In contrast to previous studies, eels in BW habitats showed no significant difference of diet and isotopic niche with silvering stages and total length, but rather a spatial difference between salinity gradients and estuaries. Also, it is considered that silver eels do not feed during migration phase (i.e. FIV, FV and MII) [7, 96, 97]. Yet, our results indicate that of the 20 silver eels analysed in this study, only 9 had empty gut contents with high δ^13^C values (greater than -22‰) suggesting a SW and BW influence, except for 2 individuals that presented depleted δ^13^C values (-33 and -31‰) may have stopped feeding in FW habitats. Indeed, it has been observed that silver eels during the early part of the migration phase (i.e. before the coastal part [98]) may temporarily stop the migration phase and resume feeding, especially when lipid reserves are insufficient (< 20% [99, 100]) to reach the Sargasso Sea [101].

### Spatial difference in dietary and isotopic niche

Spatial differences in diet were measured in European eels between salinity habitats, with eels in FW habitats feeding mainly on crustaceans and insects and shifting to macrozoobenthos and fish in SW and BW habitats [10]. The present study suggests that a difference in diet may also occur between BW habitats such as estuaries, where eels in the smallest of the six study estuaries (i.e. the Slack, the Wimereux and the Liane estuary) feed mainly on Actinopterygii rather than Malacostraca. The overlap indices confirm the distinct spatial differences in eel diet and also revealed a high overlap (> 60%) in diet between estuaries of similar environment. Spatial differences in diet have been described in other studies [102], who showed that eels feed on macrozoobenthos in Lake Vallum in Denmark, whereas they are piscivorous in Lake Großer Vätersee in Germany. This difference can be explained, not specially by the particular habitats (i.e. SW, BW or FW) occupied by the eels, but rather by the availability of macrozoobenthos [88]. Indeed, several studies (e.g. [7, 79, 83, 102]) have established a positive correlation between the diet composition of eels and the availability of potential macrozoobenthos prey. Eels have a preference for macrozoobenthos food source, except when these prey are in low abundance, the eels will shift to a piscivorous diet [102]. Our results based on SIA revealed a lower TP in eels from the largest estuaries, and may indicate a high dependence on Malacostraca rather than Actinopterygii. These results are coherent with the relationship found between the diet composition and isotopic niche of eels, %IRI in Actinopterygii in the diet and TP showed a positive correlation. The large estuaries selected for this study are composed of a large macro-crustacean and fish community [21], compared to the smaller estuaries (i.e. the Slack, the Wimereux and the Liane), which are in general mainly dominated by the fish community (R. Amara, Unpublished data). The high carbon isotope variations in the larger estuaries showed a clear separation between salinity gradients due to greater distance between stations, suggesting that the larger estuaries are likely to provide a wider niche [103]. The stable isotope values showed an enrichment from the lower part of the estuary to the upper depending on the proximity of the eels to the freshwater inflow or the tide influence.

### Biological and environmental influences on the feeding ecology of eel

Both RDA and variance partitioning indicated that eel dietary and isotopic niche variability could be related mainly to environmental differences between six estuaries. No variation of diet composition and stable isotopic with increasing total length and silvering stages were observed in this study. Spatial diet composition and stable isotopic variability was clearly demonstrated by GCA and SIA, with a wider trophic niche composed of lower TP prey (i.e. mainly from Malacostraca) rather on the larger estuaries. The large surface area of the estuary, the high connectivity with the marine environment and the predominantly sandy sediment result in a higher density and diversity of marine macrozoobenthos in the larger estuaries compared to the smaller ones. The presence of dykes, dams or harbours reduces access to habitats and food sources for the fish species [104], particularly in large estuaries where human activities are more important than in small ones, and therefore alter the fitness and reduce the growth of eels (e.g. [105–107]). However, the high trophic plasticity of eels allows them to occupy habitats that maximise their fitness [108]. In addition, the consumption of prey at higher TP provides more energy and potentially maintains high growth. Feeding of Actinopterygii seems to be more favourable from a trophic viewpoint, as Malacostraca contains much less lipids compared to Actinopterygii [109]. BW habitats are regularly considered less advantageous for eel growth and fitness compared to FW habitats [13]. Even if there are contradictory observations (e.g. [15, 110]), high macrozoobenthos prey availability in estuaries would allow good feeding activity to maximise eel growth and fitness, and thus enable fast maturation and reproductive success [13].

## Conclusion

The combination of gut content and stable isotopes analyses have made it possible to characterize the feeding ecology of the European eels in the BW habitats and to compare the dietary and isotopic niche between salinity gradients and six estuaries. The present study demonstrated that the eels fed on a variety of typical BW prey, mainly Malacostraca and Actinopterygii prey. These results reinforce the argument that part of the eel population may reside in estuaries, and highlight the important role that these estuarine habitats can provide for the eel life cycle. Both approaches led to the same pattern concluding that differences in the diet of estuarine eels exist between larger and smaller estuaries. This difference corresponds to the variation in the availability of macrozoobenthos prey which depends on the estuarine environmental conditions (i.e. surface area, tides action, sediments types), potentially reinforced by the feeding opportunism of eels. This difference in prey availability for eels could potentially affect their condition and growth rate.

Our results suggest that the two approaches of gut content and stable isotopes analyses are complementary and essential to characterise the feeding ecology of eels, one reflecting the diet composition and the other the trophic structure. A complementary approach based on condition indices could be used to test the hypothesis of an impact on condition and growth in relation to diet. Such future research will improve our understanding of the development of eels in BW habitats and fluctuations in population fitness [12], considering their physiological and nutritional conditions. It will provide a better understanding of the functioning and quality of estuaries for eels, which is necessary for better management and protection of this species.

## Supporting information

S1 Table Mean δ^15^N values of sediment organic matter (SOM) inside the Canche [54] and the Somme [55] estuaries at different salinity gradients (i.e. lower, middle, upper) and seasons (i.e. winter, spring, summer and autumn) used as baseline resource to calculate the trophic positions following Eq (4). (DOCX)

## References

[1] Als TD, Hansen MM, Maes GE, Castonguay M, Riemann L, Aarestrup K, et al. All roads lead to home: panmixia of European eel in the Sargasso Sea: panmixia of European eel in the Sargasso Sea. Mol Ecol. 2011;20: 1333–1346. doi: 10.1111/j.1365-294X.2011.05011.x 21299662

[2] Dannewitz J, Maes GE, Johansson L, Wickström H, Volckaert FAM, Järvi T. Panmixia in the European eel: a matter of time…. Proc R Soc B Biol Sci. 2005;272: 1129–1137. doi: 10.1098/rspb.2005.3064 16024374 PMC1559815

[3] Daverat F, Limburg K, Thibault I, Shiao J, Dodson J, Caron F, et al. Phenotypic plasticity of habitat use by three temperate eel species, *Anguilla anguilla*, *A*. *japonica* and *A*. *rostrata*. Mar Ecol Prog Ser. 2006;308: 231–241. doi: 10.3354/meps308231

[4] Marohn L, Jakob E, Hanel R. Implications of facultative catadromy in *Anguilla anguilla*. Does individual migratory behaviour influence eel spawner quality? J Sea Res. 2013;77: 100–106. doi: 10.1016/j.seares.2012.10.006

[5] Tzeng WN, Wang CH, Wickström H, Reizenstein M. Occurrence of the semi-catadromous European eel *Anguilla anguilla* in the Baltic Sea. Mar Biol. 2000;137: 93–98. doi: 10.1007/s002270000330

[6] Moriarty C. The Yellow Eel. In: Aida K, Tsukamoto K, Yamauchi K, editors. Eel Biology. Tokyo: Springer Japan; 2003. pp. 89–105. doi: 10.1007/978-4-431-65907-5_7

[7] Tesch FW. The Eel. Blackwell Science. Oxford, United Kingdom; 2003. doi: https://doi.org/10.1111/j.0022-1112.2004.00509a.x

[8] Correia MJ, Costa JL, Antunes C, De Leo G, Domingos I. The decline in recruitment of the European eel: new insights from a 40-year-long time-series in the Minho estuary (Portugal). ICES J Mar Sci. 2018;75: 1975–1983. doi: 10.1093/icesjms/fsy073

[9] Morais P, Daverat F. An Introduction to Fish Migration. CRC Press. Boca Raton, Florida (USA); 2016. ⟨hal-02603390⟩

[10] Harrod C, Grey J, McCarthy TKieran, Morrissey M. Stable isotope analyses provide new insights into ecological plasticity in a mixohaline population of European eel. Oecologia. 2005;144: 673–683. doi: 10.1007/s00442-005-0161-x 16025352

[11] Arai T. Ecology and evolution of migration in the freshwater eels of the genus *Anguilla* Schrank, 1798. Heliyon. 2020;6: e05176. doi: 10.1016/j.heliyon.2020.e05176 33083623 PMC7553983

[12] Arai T, Chino N. Diverse migration strategy between freshwater and seawater habitats in the freshwater eel genus *Anguilla*. J Fish Biol. 2012;81: 442–455. doi: 10.1111/j.1095-8649.2012.03353.x 22803719

[13] Gross MR, Coleman RM, McDowall RM. Aquatic Productivity and the Evolution of Diadromous Fish Migration. Science. 1988;239: 1291–1293. doi: 10.1126/science.239.4845.1291 17833216

[14] Daverat F, Beaulaton L, Poole R, Lambert P, Wickström H, Andersson J, et al. One century of eel growth: changes and implications: One century of eel growth. Ecol Freshw Fish. 2012;21: 325–336. doi: 10.1111/j.1600-0633.2011.00541.x

[15] Daverat F, Tomás J. Tactics and demographic attributes in the European eel *Anguilla anguilla* in the Gironde watershed, SW France. Mar Ecol Prog Ser. 2006;307: 247–257. doi: 10.3354/meps307247

[16] Patey G, Couillard CM, Drouineau H, Verreault G, Pierron F, Lambert P, et al. Early back-calculated size-at-age of Atlantic yellow eels sampled along ecological gradients in the Gironde and St. Lawrence hydrographical systems. Can J Fish Aquat Sci. 2018;75: 1270–1279. doi: 10.1139/cjfas-2017-0025

[17] Jessop B, Shiao J, Iizuka Y, Tzeng W. Migratory behaviour and habitat use by American eels *Anguilla rostrata* as revealed by otolith microchemistry. Mar Ecol Prog Ser. 2002;233: 217–229. doi: 10.3354/meps233217

[18] Melia P, Bevacqua D, Crivelli AJ, De Leo GA, Panfili J, Gatto M. Age and growth of *Anguilla anguilla* in the Camargue lagoons. J Fish Biol. 2006;68: 876–890. doi: 10.1111/j.0022-1112.2006.00975.x

[19] Yokouchi K, Aoyama J, Miller MJ, McCarthy TK, Tsukamoto K. Depth distribution and biological characteristics of the European eel *Anguilla anguilla* in Lough Ennell, Ireland. J Fish Biol. 2009;74: 857–871. doi: 10.1111/j.1095-8649.2008.02167.x 20735604

[20] Amara R. Seasonal ichthyodiversity and growth patterns of juvenile flatfish on a nursery ground in the Southern Bight of the North Sea (France). Environ Biol Fishes. 2003;67: 191–201. doi: 10.1023/A:1025646622066

[21] Selleslagh J, Amara R, Laffargue P, Lesourd S, Lepage M, Girardin M. Fish composition and assemblage structure in three Eastern English Channel macrotidal estuaries: A comparison with other French estuaries. Estuar Coast Shelf Sci. 2009;81: 149–159. doi: 10.1016/j.ecss.2008.10.008

[22] Elliott M, Hemingway K, editors. Fishes in estuaries. John Wiley&Sons. Fishes in estuaries. John Wiley&Sons. Oxford: Blackwell Science; 2002.

[23] McLusky DS, Elliott M. The Estuarine Ecosystem. Oxford University Press; 2004. doi: 10.1093/acprof:oso/9780198525080.001.0001

[24] Jacoby DMP, Casselman JM, Crook V, DeLucia M-B, Ahn H, Kaifu K, et al. Synergistic patterns of threat and the challenges facing global anguillid eel conservation. Glob Ecol Conserv. 2015;4: 321–333. doi: 10.1016/j.gecco.2015.07.009

[25] Righton D, Piper A, Aarestrup K, Amilhat E, Belpaire C, Casselman J, et al. Important questions to progress science and sustainable management of anguillid eels. Fish Fish. 2021;22: 762–788. doi: 10.1111/faf.12549

[26] Arai T, Chino N, Le DQ. Migration and habitat use of the tropical eels *Anguilla marmorata* and *A*. *bicolor pacifica* in Vietnam. Aquat Ecol. 2013;47: 57–65. doi: 10.1007/s10452-012-9424-x

[27] Kotake A, Okamura A, Yamada Y, Utoh T, Arai T, Miller M, et al. Seasonal variation in the migratory history of the Japanese eel *Anguilla japonica* in Mikawa Bay, Japan. Mar Ecol Prog Ser. 2005;293: 213–221. doi: 10.3354/meps293213

[28] Hynes HBN. The food of fresh-water sticklebacks (*Gasterosteus aculeatus* and *Pygosteus pungitius*), with a review of methods used in studies of the food of fishes. J Anim Ecol. 1950;19: 36–58. doi: 10.2307/1570

[29] Hyslop EJ. Stomach contents analysis-a review of methods and their application. J Fish Biol. 1980;17: 411–429. doi: 10.1111/j.1095-8649.1980.tb02775.x

[30] Parker PL. The biogeochemistry of the stable isotopes of carbon in a marine bay. Geochim Cosmochim Acta. 1964;28: 1155–1164. doi: 10.1016/0016-7037(64)90067-5

[31] DeNiro MJ, Epstein S. Influence of diet on the distribution of carbon isotopes in animals. Geochim Cosmochim Acta. 1978;42: 495–506. doi: 10.1016/0016-7037(78)90199-0

[32] Minagawa M, Wada E. Stepwise enrichment of ^15^N along food chains: Further evidence and the relation between δ^15^N and animal age. Geochim Cosmochim Acta. 1984;48: 1135–1140. doi: 10.1016/0016-7037(84)90204-7

[33] Costa JL, Assis CA, Almeida PR, Moreira FM, Costa MJ. On the food of the European eel, *Anguilla anguilla* (L.), in the upper zone of the Tagus estuary, Portugal. J Fish Biol. 1992;41: 841–850. doi: 10.1111/j.1095-8649.1992.tb02712.x

[34] Deelder CL. Synopsis of biological data on the eel, *Anguilla anguilla* (Linnaeus, 1758). FAO Fish Synop No 80 Revis 1. 1984;1: 1–73.

[35] Moriarty C. Studies of the eel *Anguilla anguilla* in Ireland 1. In the *lakes of the Corrib System*. *Station Off*. 1972; 20.

[36] Capoccioni F, Leone C, Giustini F, Brilli M, Buttazzoni L, Hanel R, et al. δ^13^C and δ^15^N in yellow and silver eels (*Anguilla anguilla*, 1758) from different Mediterranean local stocks and their variation with body size and growth. Mar Freshw Res. 2021;72: 1208–1219. doi: 10.1071/MF20144

[37] Layman CA, Winemiller KO, Arrington DA. Describing a species-rich river food web using stable isotopes, stomach contents, and functional experiments. Dynamic Food Webs. Elsevier; 2005. pp. 395–406. doi: 10.1016/B978-012088458-2/50037-0

[38] Fry B. Conservative Mixing of Stable Isotopes across Estuarine Salinity Gradients: A Conceptual Framework for Monitoring Watershed Influences on Downstream Fisheries Production. Estuaries. 2002;25: 264–271.

[39] Selleslagh J, Amara R. Inter-season and interannual variations in fish and macrocrustacean community structure on a eastern English Channel sandy beach: Influence of environmental factors. Estuar Coast Shelf Sci. 2008;77: 721–730. doi: 10.1016/j.ecss.2007.11.004

[40] Nicolas D, Lobry J, Le Pape O, Boët P. Functional diversity in European estuaries: Relating the composition of fish assemblages to the abiotic environment. Estuar Coast Shelf Sci. 2010;88: 329–338. doi: 10.1016/j.ecss.2010.04.010

[41] Selleslagh J, Lesourd S, Amara R. Comparison of macrobenthic assemblages of three fish estuarine nurseries and their importance as foraging grounds. J Mar Biol Assoc U K. 2011;92: 85–97. doi: 10.1017/S0025315411000336

[42] Durif C, Dufour S, Elie P. The silvering process of *Anguilla anguilla*: a new classification from the yellow resident to the silver migrating stage. J Fish Biol. 2005;66: 1025–1043. doi: 10.1111/j.0022-1112.2005.00662.x

[43] Durif C, Guibert A, Elie P. Morphological discrimination of the silvering stages of the European eel. Eels at the Edge Science, Status, and Conservation Concerns. 2009. pp. 103–111.

[44] Martin J. Les invertébrés marins du golfe de Gascogne à la Manche orientale. Editions Quae; 2011.

[45] Lincoln RJ. British marine amphipoda: Gammaridea. British Museum (Natural History); 1979.

[46] Birmingham M, Heimdal D, Hubbard T, Krier K, Leopold R, Luzier J, et al. Benthic Macroinvertebrate Key. 2005 p. 27. http://www.iowadnr.gov/Portals/idnr/uploads/watermonitoring/iowater/Publications/BMIKey2Edvmay05.pdf

[47] Quero J-C, Vayne J-J. Clé de détermination des poissons marins de l’Atlantique du nord-est (entre le 80° et le 30° parallèle nord)-II. Pleuronectiformes. 1979 p. 41. http://archimer.ifremer.fr/doc/00060/17101/

[48] Keith P, Allardi J. Atlas des poissons d’eau douce de France. Paris; 2001.

[49] Berg J. Discussion of methods of investigating the food of fishes, with reference to a preliminary study of the prey of *Gobiusculus flavescens* (Gobiidae). Mar Biol. 1979;50: 263–273. doi: 10.1007/BF00394208

[50] Cortés E. A critical review of methods of studying fish feeding based on analysis of stomach contents: application to elasmobranch fishes. Can J Fish Aquat Sci. 1997;54: 726–738. doi: 10.1139/f96-316

[51] Pinkas L. Food habits study. Fish Bull. 1971;152: 5–10.

[52] Chouvelon T, Spitz J, Cherel Y, Caurant F, Sirmel R, Mèndez-Fernandez P, et al. Inter-specific and ontogenic differences in δ^13^C and δ^15^N values and Hg and Cd concentrations in cephalopods. Mar Ecol Prog Ser. 2011;433: 107–120. doi: 10.3354/meps09159

[53] Cabana G, Rasmussen JB. Comparison of aquatic food chains using nitrogen isotopes. Proc Natl Acad Sci. 1996;93: 10844–10847. doi: 10.1073/pnas.93.20.10844 8855268 PMC38243

[54] Bouaziz R, Le Loc’h F, Rolet C, Veillet G, Munaron JM, Rabhi K, et al. Structure and seasonal variability in fish food webs in a small macrotidal estuary (Canche estuary, Eastern English Channel) based on stable carbon and nitrogen isotope analysis. Reg Stud Mar Sci. 2021;44: 101694. doi: 10.1016/j.rsma.2021.101694

[55] Meirland A, Rybarczyk H, Catterou M. COMORES: Cycle et Origine de la Matière Organique du Réseau trophique de l’Estuaire de la Somme. Action 9: Isotopes et acides gras. 2013 p. 125p. Report No.: n° 13–047.

[56] Post DM. Using stable isotopes to estimate trophic position: models, methods, and assumptions. Ecology. 2002;83: 703–718. doi: 10.1890/0012-9658(2002)083[0703:USITET]2.0.CO;2

[57] Layman CA, Arrington DA, Montaña CG, Post DM. Can stable isotope ratios provide for community‐wide measures of trophic structure? Ecology. 2007;88: 42–48. doi: 10.1890/0012-9658(2007)88[42:csirpf]2.0.co;2 17489452

[58] Jackson AL, Inger R, Parnell AC, Bearhop S. Comparing isotopic niche widths among and within communities: SIBER—Stable Isotope Bayesian Ellipses in R: Bayesian isotopic niche metrics. J Anim Ecol. 2011;80: 595–602. doi: 10.1111/j.1365-2656.2011.01806.x 21401589

[59] Anderson MJ. Permutation tests for univariate or multivariate analysis of variance and regression. Can J Fish Aquat Sci. 2001;58: 626–639. doi: 10.1139/f01-004

[60] Benjamini Y, Hochberg Y. Controlling the False Discovery Rate: A Practical and Powerful Approach to Multiple Testing. J R Stat Soc Ser B Methodol. 1995;57: 289–300. doi: 10.1111/j.2517-6161.1995.tb02031.x

[61] Oksanen J, Blanchet FG, Kindt R, Legendre P, Minchin PR, O’Hara RB, et al. Package ‘vegan.’ R Packag Ver. 2013;254: 20–8.

[62] Hervé M, Hervé MM. Package ‘RVAideMemoire’. R Package. 2020. https://CRAN.R-project.org/package=RVAideMemoire.

[63] Clarke KR, Gorley RN, Somerfield PJ, Warwick RM. Change in marine communities: an approach to statistical analysis and interpretation. 3rd edition. Plymouth: Primer-E Ltd.; 2014. http://plymsea.ac.uk/id/eprint/7656

[64] Whitaker D, Christman M. Package “clustsig.” R Packag Ver. 2014. http://www.douglaswhitaker.com

[65] Schoener TW. Nonsynchronous Spatial Overlap of Lizards in Patchy Habitats. 1970; 12.

[66] Mathur D. Food Habits and Competitive Relationships of the Bandfin Shiner in Halawakee Creek, Alabama. Am Midl Nat. 1977;97: 89–100. doi: 10.2307/2424687

[67] Zaret TM, Rand AS. Competition in Tropical Stream Fishes: Support for the Competitive Exclusion Principle. Ecology. 1971;52: 336–342. doi: 10.2307/1934593

[68] Swanson HK, Lysy M, Power M, Stasko AD, Johnson JD, Reist JD. A new probabilistic method for quantifying *n*-dimensional ecological niches and niche overlap. Ecology. 2015;96: 318–324. doi: 10.1890/14-0235.1 26240852

[69] Lysy M, Stasko AD, Swanson HK. nicheROVER: (niche) (r)egion and niche (over)lap metrics for multidimensional ecological niches. R package version 1.0. 2014. https://CRAN.R-project.org/package=nicheROVER

[70] Cucherousset J, Acou A, Blanchet S, Britton JR, Beaumont WRC, Gozlan RE. Fitness consequences of individual specialisation in resource use and trophic morphology in European eels. Oecologia. 2011;167: 75–84. doi: 10.1007/s00442-011-1974-4 21455773

[71] Lomolino MV. Ecology’s most general, yet protean 1 pattern: the species-area relationship. J Biogeogr. 2000;27: 17–26. doi: 10.1046/j.1365-2699.2000.00377.x

[72] Monaco ME, Lowery TA, Emmett RL. Assemblages of U.S. West Coast Estuaries Based on the Distribution of Fishes. J Biogeogr. 1992;19: 251. doi: 10.2307/2845450

[73] Pratt DR, Lohrer AM, Pilditch CA, Thrush SF. Changes in Ecosystem Function Across Sedimentary Gradients in Estuaries. Ecosystems. 2014;17: 182–194. doi: 10.1007/s10021-013-9716-6

[74] Sousa R, Dias S, Freitas V, Antunes C. Subtidal macrozoobenthic assemblages along the River Minho estuarine gradient (north-west Iberian Peninsula). Aquat Conserv Mar Freshw Ecosyst. 2008;18: 1063–1077. doi: 10.1002/aqc.871

[75] Lake JL, McKinney RA, Osterman FA, Pruell RJ, Kiddon J, Ryba SA, et al. Stable nitrogen isotopes as indicators of anthropogenic activities in small freshwater systems. Can J Fish Aquat Sci. 2001;58: 870–878. doi: 10.1139/cjfas-58-5-870

[76] Borcard D, Gillet F, Legendre P. Numerical Ecology with R. New York: Springer; 2011. doi: 10.1007/978-1-4419-7976-6

[77] Pasquaud S, Elie P, Jeantet C, Billy I, Martinez P, Girardin M. A preliminary investigation of the fish food web in the Gironde estuary, France, using dietary and stable isotope analyses. Estuar Coast Shelf Sci. 2008;78: 267–279. doi: 10.1016/j.ecss.2007.12.014

[78] Bowman RE. Effect of regurgitation on stomach content data of marine fishes. Environ Biol Fishes. 1986;16: 171–181. doi: 10.1007/BF00005169

[79] Moore JW, Moore IA. The basis of food selection in some estuarine fishes. Eels, *Anguilla anguilla* (L.), whiting, *Merlangius merlangus* (L.), sprat, *Sprattus sprattus* (L.) and stickleback, Gasterosteus aculeatus L. J Fish Biol. 1976;9: 375–390. doi: 10.1111/j.1095-8649.1976.tb04686.x

[80] Degani G, Levanon D. The influence of low density on food adaptation, cannibalism and growth of eels (*Anguilla anguilla* (L.)). Bamidgeh. 1983;35: 53–60.

[81] Imbert H, Arrowsmith R, Dufour S, Elie P. Relationships between locomotor behavior, morphometric characters and thyroid hormone levels give evidence of stage-dependent mechanisms in European eel upstream migration. Horm Behav. 2008;53: 69–81. doi: 10.1016/j.yhbeh.2007.06.011 17950736

[82] Knights B. Agonistic behaviour and growth in the European eel, *Anguilla anguilla* L., in relation to warm-water aquaculture. J Fish Biol. 1987;31: 265–276. doi: 10.1111/j.1095-8649.1987.tb05230.x

[83] Bouchereau J-L, Marques C, Pereira P, Guélorget O, Vergne Y. Food of the European eel *Anguilla anguilla* in the Mauguio lagoon (Mediterranean, France). Acta Adriat. 2009;50: 159–170.

[84] Parzanini C, Arts MT, Rohtla M, Koprivnikar J, Power M, Skiftesvik AB, et al. Feeding Habitat and Silvering Stage Affect Lipid Content and Fatty Acid Composition of European Eel *Anguilla anguilla* Tissues. J Fish Biol. 2021; jfb.14815. doi: 10.1111/jfb.14815 34060093

[85] Vasconi M, Lopez A, Galimberti C, Moreno Rojas JM, Muñoz Redondo JM, Bellagamba F, et al. Authentication of farmed and wild european eel (*Anguilla anguilla*) by fatty acid profile and carbon and nitrogen isotopic analyses. Food Control. 2019;102: 112–121. doi: 10.1016/j.foodcont.2019.03.004

[86] Fry B. Food web structure on Georges Bank from stable C, N, S isotopic compositions. Limnol Oceanogr. 1988;33: 1182–1190. doi: 10.4319/lo.1988.33.5.1182

[87] Day JW, Yáñez-Arancibia A, Kemp WM, Crump BC. Introduction to Estuarine Ecology. In: Day JW, Crump BC, Kemp WM, Yáñez-Arancibia A, editors. Estuarine Ecology. Hoboken, NJ, USA: John Wiley & Sons, Inc.; 2012. pp. 1–19. doi: 10.1002/9781118412787.ch1

[88] Arai T. Biology and Ecology of Anguillid Eels. CRC Press. 2016.

[89] De Nie HW. A note on the significance of larger bivalve molluscs (*Anodonta* spp. and *Dreissena* sp.) in the food of the eel (*Anguilla anguilla*) in Tjeukemeer. Hydrobiologia. 1982;95: 307–310. doi: 10.1007/BF00044491

[90] Ezzat AE, El-Seraffy SS. Food of *Anguilla anguilla* in Lake Manzalah, Egypt. Mar Biol. 1977;41: 287–291. doi: 10.1007/BF00394917

[91] Harrod C, Grey J. Isotopic variation complicates analysis of trophic relations within the fish community of Plußsee: a small, deep, stratifying lake. Arch Für Hydrobiol. 2006;167: 281–299. doi: 10.1127/0003-9136/2006/0167-0281

[92] De Meyer J, Christiaens J, Adriaens D. Diet-induced phenotypic plasticity in European eel (*Anguilla anguilla*). J Exp Biol. 2016;219: 354–363. doi: 10.1242/jeb.131714 26847560

[93] Verhelst P, De Meyer J, Reubens J, Coeck J, Goethals P, Moens T, et al. Unimodal head-width distribution of the European eel (*Anguilla anguilla* L.) from the Zeeschelde does not support disruptive selection. PeerJ. 2018;6: e5773. doi: 10.7717/peerj.5773 30416881 PMC6225841

[94] Dörner H, Benndorf J. Piscivory by large eels on young-of-the-year fishes: its potential as a biomanipulation tool. J Fish Biol. 2003;62: 491–494. doi: 10.1046/j.1095-8649.2003.00035.x

[95] Ryan PA. Seasonal and size-related changes in the food of the short-finned eel, *Anguilla australis* in Lake Ellesmere, Canterbury, New Zealand. Environ Biol Fishes. 1986;15: 47–58. doi: 10.1007/BF00005388

[96] Chow S, Kurogi H, Katayama S, Ambe D, Okazaki M, Watanabe T, et al. Japanese eel Anguilla japonica do not assimilate nutrition during the oceanic spawning migration: evidence from stable isotope analysis. Mar Ecol Prog Ser. 2010;402: 233–238. doi: 10.1007/s10126-010-9301-3 20535520

[97] Fricke H, Kaese R. Tracking of Artificially Matured Eels (*Anguilla anguilla*) in the Sargasso Sea and the Problem of the Eel’s Spawning Site. 1995; 5.

[98] Pankhurst NW, Sorensen PW. Degeneration of the alimentary tract in sexually maturing European *Anguilla anguilla* (L.) and American eels *Anguilla rostrata* (LeSueur). Can J Zool. 1984;62: 1143–1149. doi: 10.1139/z84-165

[99] Belpaire CGJ, Goemans G, Geeraerts C, Quataert P, Parmentier K, Hagel P, et al. Decreasing eel stocks: survival of the fattest? Ecol Freshw Fish. 2009;18: 197–214. doi: 10.1111/j.1600-0633.2008.00337.x

[100] Van den Thillart GEEJ, Palstra A, Van Ginneken V. Simulated migration of European silver eel; swim capacity and cost of transport. J Mar Sci Technol. 2007;15: 1–16. doi: 10.1007/s10695-010-9397-4 20390348 PMC2923712

[101] Svedäng H, Wickström H. Low fat contents in female silver eels: indications of insufficient energetic stores for migration and gonadal development. J Fish Biol. 1997;50: 475–486. doi: 10.1111/j.1095-8649.1997.tb01943.x

[102] Dörner H, Skov C, Berg S, Schulze T, Beare DJ, Van der Velde G. Piscivory and trophic position of *Anguilla anguilla* in two lakes: importance of macrozoobenthos density. J Fish Biol. 2009;74: 2115–2131. doi: 10.1111/j.1095-8649.2009.02289.x 20735691

[103] Haegeman B, Loreau M. A mathematical synthesis of niche and neutral theories in community ecology. J Theor Biol. 2011;269: 150–165. doi: 10.1016/j.jtbi.2010.10.006 20946903

[104] Baudoin J-M, Burgun V, Chanseau M, Larinier M, Ovidio M, Sremski W, et al. Évaluer le franchissement des obstacles par les poissons: principes et méthodes: informations sur la continuité écologique, ICE. Vincennes: ONEMA; 2014.

[105] Cairns DK, Secor DA, Morrison WE, Hallett JA. Salinity-linked growth in anguillid eels and the paradox of temperate-zone catadromy. J Fish Biol. 2009;74: 2094–2114. doi: 10.1111/j.1095-8649.2009.02290.x 20735690

[106] Geeraerts C, Belpaire C. The effects of contaminants in European eel: a review. Ecotoxicology. 2010;19: 239–266. doi: 10.1007/s10646-009-0424-0 19806452

[107] Simpson SD, Purser J, Radford AN. Anthropogenic noise compromises antipredator behaviour in European eels. Glob Change Biol. 2015;21: 586–593. doi: 10.1111/gcb.12685 25098970

[108] Edeline E. Adaptive phenotypic plasticity of eel diadromy. Mar Ecol Prog Ser. 2007;341: 229–232. doi: 10.3354/meps341229

[109] Helfman GS, Winkelman DL. Energy Trade-Offs and Foraging Mode Choice in American Eels. Ecology. 1991;72: 310–318. doi: 10.2307/1938924

[110] Arai T, Kotake A, McCarthy TK. Habitat use by the European eel *Anguilla anguilla* in Irish waters. Estuar Coast Shelf Sci. 2006;67: 569–578. doi: 10.1016/j.ecss.2006.01.001

