# Supplementary material for: Role of estuarine habitats for the feeding ecology of the European eel (Anguilla anguilla L.)
Source: PLoS One. 2022 Jul 6;17(7):e0270348. doi: 10.1371/journal.pone.0270348 (PMC9261484; doi:10.1371/journal.pone.0270348)
Supplement: S1 Table — (DOCX) [file pone.0270348.s001.docx]

**S1 Table. Mean δ^15^N values of sediment organic matter (SOM) inside the Canche [47] and the Somme [48] estuaries at different salinity gradients (i.e. lower, middle, upper) and seasons (i.e. winter, spring, summer and autumn) used as baseline resource to calculate the trophic positions following Eq. (4).**

| **Estuary** | **Salinity gradients** | **Winter** | **Spring** | **Summer** | **Autumn** |
| --- | --- | --- | --- | --- | --- |
| **Canche** | **Lower** | 6.07 | 4.67 | 4.67 | 6.07 |
|  | **Middle** | 7.19 | 5.39 | 5.39 | 7.19 |
|  | **Upper** | 6.00 | 5.46 | 5.46 | 6.00 |
| **Somme** | **Lower** | 8.07 | 8.32 | 8.53 | 7.67 |
|  | **Middle** | 6.93 | 7.73 | 8.05 | 7.59 |
|  | **Upper** | 7.33 | 8.98 | 8.65 | 7.41 |
